# Supplementary material for: Temporally organized representations of reward and risk in the human brain
Source: Nat Commun. 2024 Mar 9;15:2162. doi: 10.1038/s41467-024-46094-1 (PMC10924934; doi:10.1038/s41467-024-46094-1)
Supplement: Supplementary file 1 — Supplementary Information [file 41467_2024_46094_MOESM1_ESM.pdf]

Supplementary materials for “Temporally organized representations of reward and risk in the human brain”

Vincent Man[1], Jeffrey Cockburn[1], Oliver Flouty[2], Phillip E. Gander[3,4,5], Masahiro Sawada[3], Christopher K. Kovach[3,6], Hiroto Kawasaki[3], Hiroyuki Oya[3,5], Matthew A. Howard III[3,5], John P. O’Doherty[1,7]

[1]Division of the Humanities and Social Sciences, California Institute of Technology, Pasadena, CA 91125, USA
[2]Department of Neurosurgery and Brain Repair, University of South Florida, Tampa, FL 33606, USA
[3]Department of Neurosurgery, University of Iowa Hospitals & Clinics, Iowa City, IA 52242, USA
[4]Department of Radiology, University of Iowa Hospitals & Clinics, Iowa City, IA 52242, USA
[5]Iowa Neuroscience Institute, University of Iowa Carver College of Medicine, Iowa City, IA 52242, USA
[6]Department of Neurosurgery, University of Nebraska Medical Center, Omaha, Nebraska 68198, USA
[7]Computation and Neural Systems, California Institute of Technology, Pasadena, CA 91125, USA

ROI	# Participants	# Contacts	# L	# R
Ant. Insula	6	14	4	10
Post. Insula	5	10	6	4
OFC	10	72	30	42
vmPFC	5	12	6	6
Frontal Pole	8	158	58	100
Putamen	4	8	0	8
Hippocampus	10	62	20	42
Amygdala	8	32	12	20
Cingulate Gyr	6	16	2	14
Supramarg. Gyr	6	30	2	28
Angular Gyr	5	14	6	8

Supplementary Table 1: Distribution of contacts per ROI. # Participants denotes the sample size for each ROI. # Contacts is the number of recording sites per ROI. # L and # R denote left and right hemisphere contact counts, respectively.

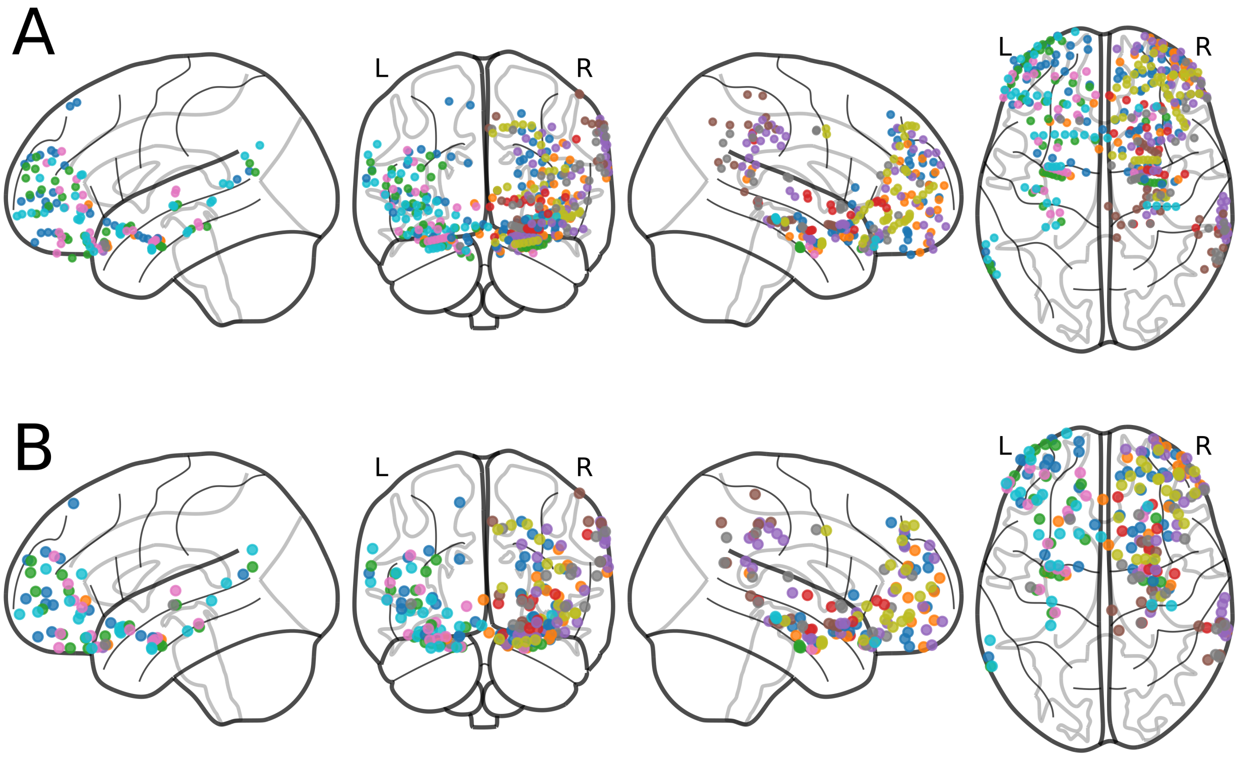

Supplementary Figure 1: Contact coordinates in original recording (A) and projected bipolar source (B) locations displayed over template space. Bipolar locations are averages across anode and cathode pair coordinates in three dimensions. Panel B is the same as Figure 1B, but organized by participant ID rather than ROI. Different colours identify distinct participants. Copyright (c) 2007 - 2023 The nilearn developers. All rights reserved. https://github.com/nilearn/nilearn. Source data are provided as a Source Data file.

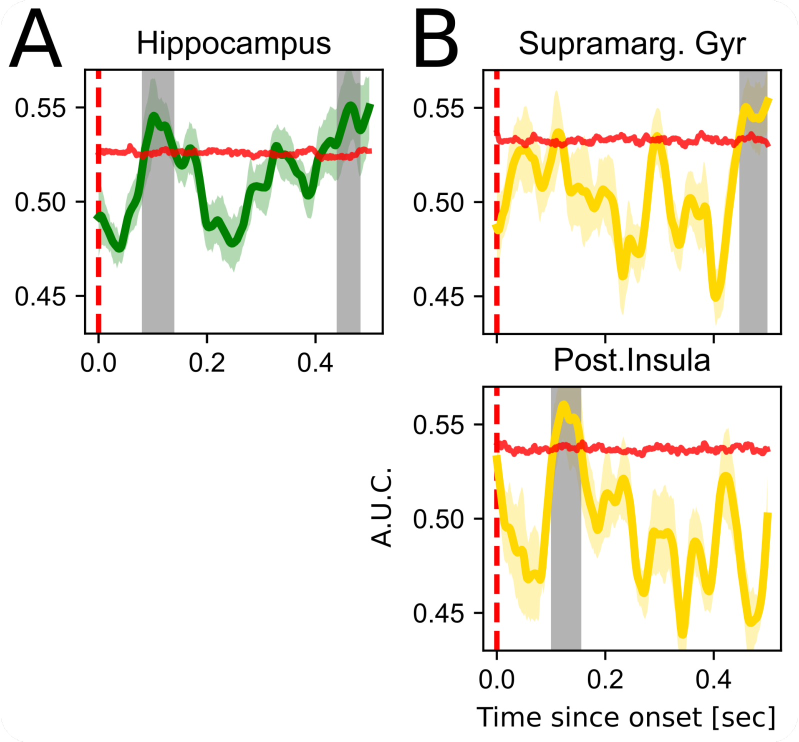

Supplementary Figure 2: Decoding of (A) expected value and B) expected risk after the offset of card 1 in exploratory ROIs. Lines depict cross-validated receiver operating characteristic (ROC) area under the curve (AUC), and shaded coloured areas show standard error in ROC AUC across folds. Horizontal red lines depict the 95th percentile of the permuted null distribution at each time point, and periods of statistical significance are shown in the shaded grey region (cluster corrected FWE < 0.05). Decoding curves are low-pass filtered (1st-order Butterworth filter at 0.1 Hz) for visualization; all statistics are conducted on non low-pass filtered data. Source data are provided as a Source Data file.

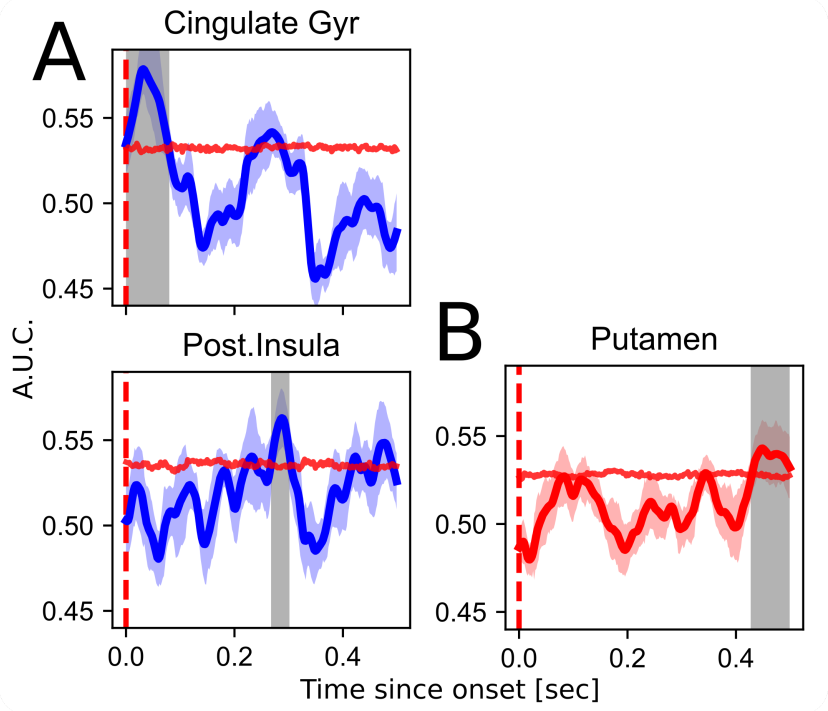

Supplementary Figure 3: Decoding of (A) reward prediction error and (B) risk prediction error in exploratory ROIs. Description follows that described in Figure S2. Source data are provided as a Source Data file.

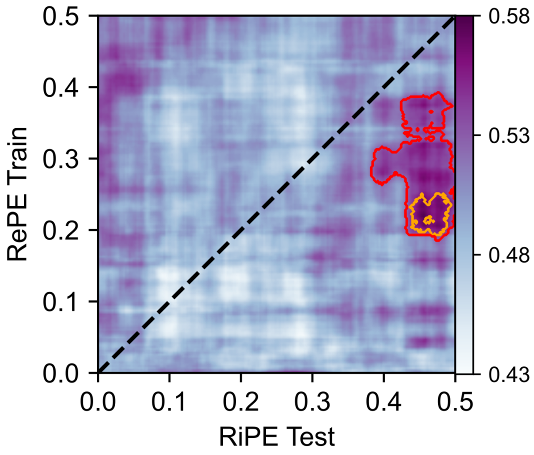

Supplementary Figure 4: Generalized decoding in anterior insula. Same as Figure 6A, but across the entire epoch after the onset of card 2 [0.0 - 0.500 sec]. Significant decoding areas are highlighted by the red (p = 0.031; one-sided non-parametric test) and orange (p = 0.003; one-sided non-parametric test) contours (both cluster corrected FWE < 0.05). Source data are provided as a Source Data file.

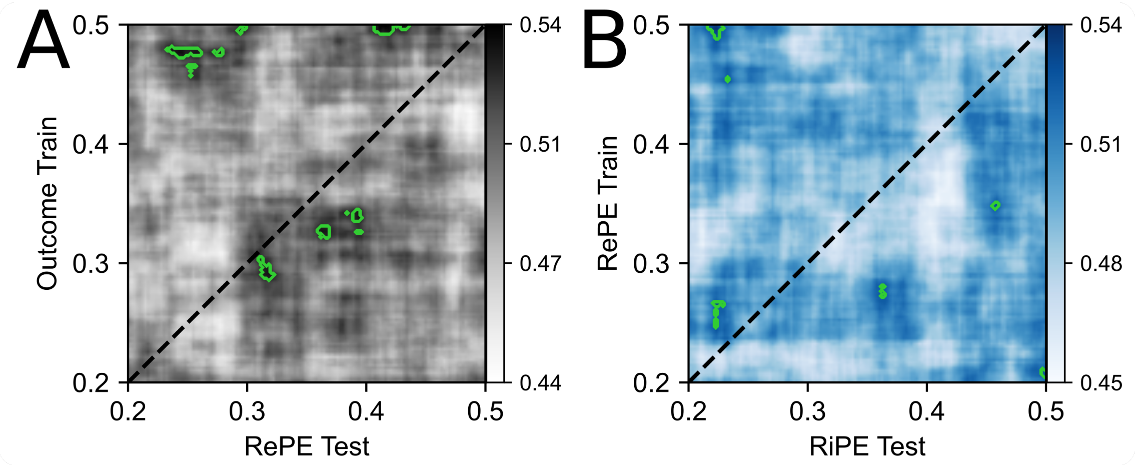

Supplementary Figure 5: Lack of generalized decoding in OFC for models (A) trained on the multivariate signal in OFC encoding outcome and tested on RePE, and (B) trained on RePE and tested on RiPE. Areas highlighted in green reflect decoding accuracies above the 95th percentile of the permuted null distribution, but for both models no cluster survived correction at FWE < 0.05. Source data are provided as a Source Data file.

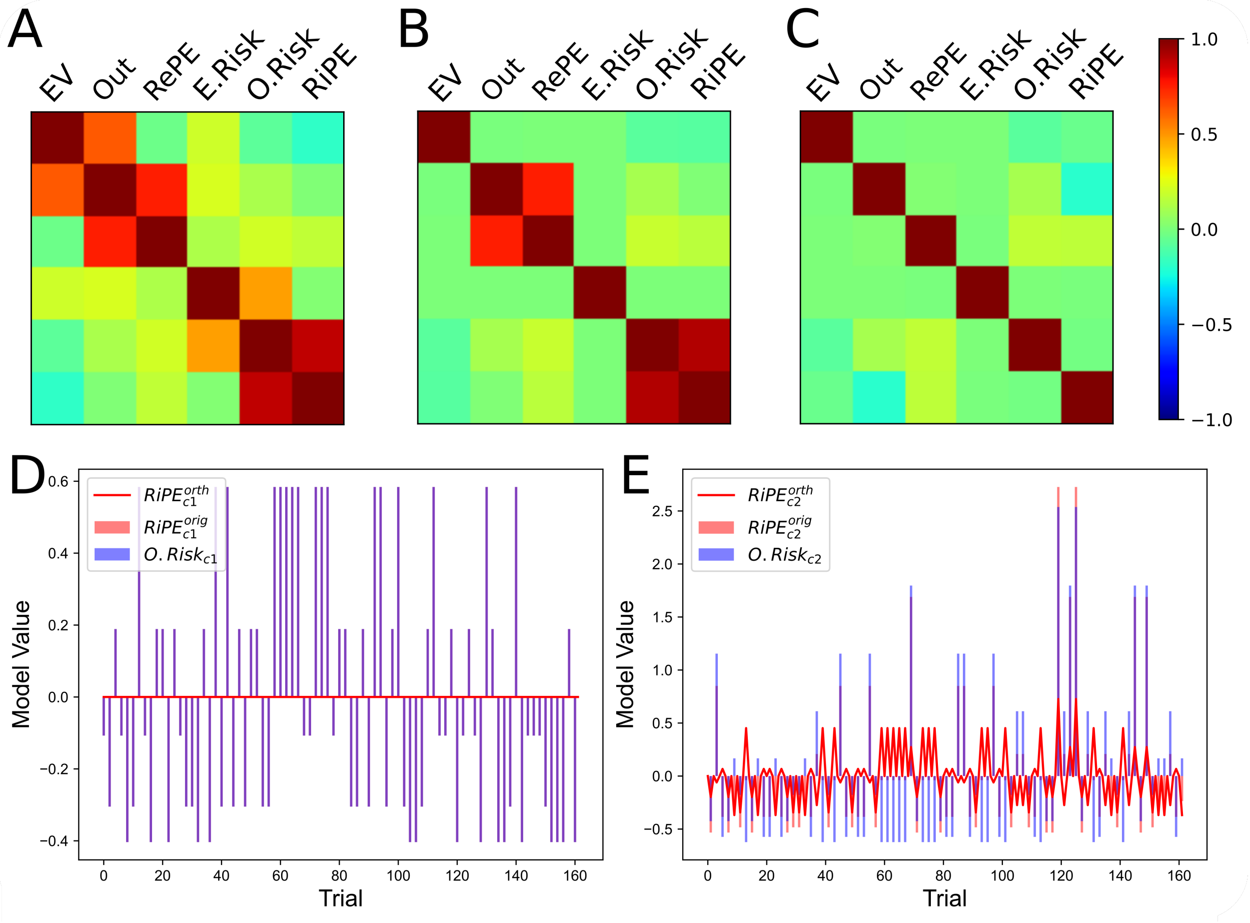

Supplementary Figure 6: Correlation between computational variables. (A) As derived directly from behavior and the normative model, there are substantial correlations between the computational variables. (B) Our expanded design matrix spaced out comptuational variables across time within a trial and situated each variable at their hypothesized occurence given the information available to the participant at that moment (e.g. outcome after the second card is presented). This approach decoupled most variables. (C) Variables that remained highly correlated after our expanded design matrix (i.e., outcome and RePE; observed risk and RiPE) were statistically orthogonalized such that the outcome and observed risk variables retained variance previously shared with RePE and RiPE, respectively. (D) The effect of statistical orthogonalization at card 1 in which shared variance between O.Risk$_{card1}$ and RiPE$_{card1}$ is given to O.Risk$_{card1}$ (blue bars); because the variables are perfectly correlated, RiPE$_{card1}$ becomes a constant (red line at 0). (E) Same as D, but for card 2. Here the variables are separable enough that RiPE$_{card2}$ after orthogonlization retains the unique variance above and beyond O.Risk$_{card2}$ (red line). Source data are provided as a Source Data file.

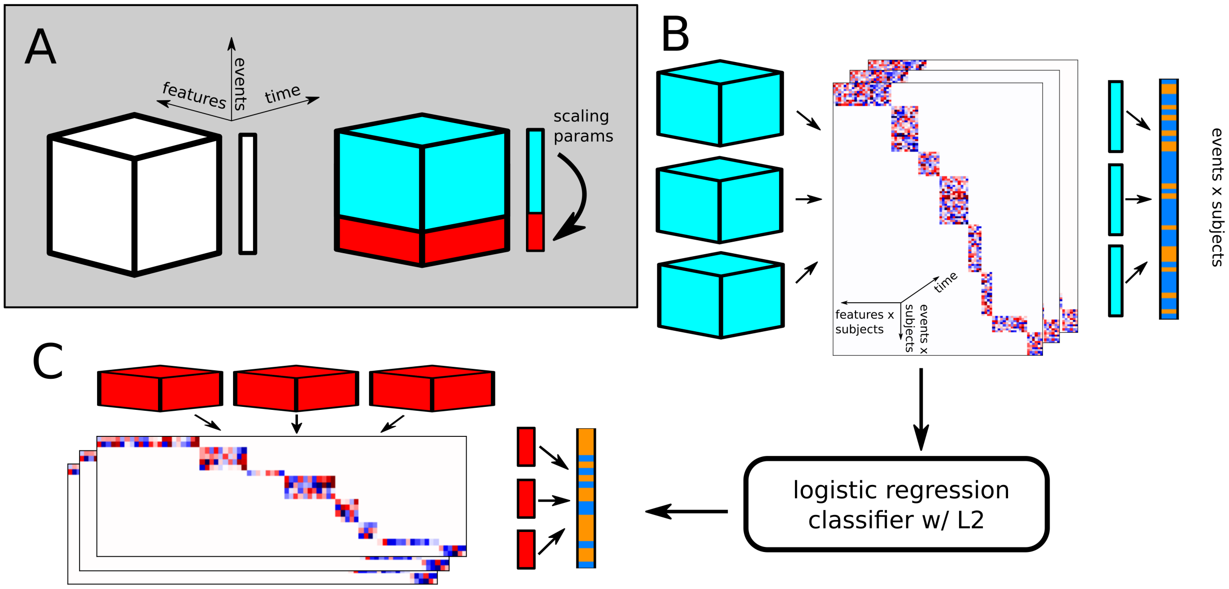

Supplementary Figure 7: Overview of pseudo-population decoding approach. (A) Left: The feature matrix for a single participant is organized along features (neural sources), events (within-trial events) and time (samples) dimensions. The target vector has the dimensionality of events. Right: The features are preprocessed and split into train (cyan) and test (red) folds within-subject. Preprocessing parameters are estimated on the training data and applied to both the train and test sets. (B) The train sets across participants are concatenated diagonally into a pseudo-population matrix, which has features x subjects, events x subjects and time dimensions, for a given ROI. Each tile in the pseudo-population concatenated matrix (middle) corresponds to the neural data from one participant. The target vectors across participants are concatenated and of events x subjects length. The concatenated feature matrix and target vector are used to train the logistic regression classifier, which is then tested on the left-out data (C) which is organized across participants similarly. A single cross-validation fold is depicted; this procedure is iterated across 10 folds and average receiver operating characteristic area under the curve (ROC AUC) is reported as the accuracy metric.

Supplementary Note 1: Prefrontal sub-region analysis

We originally defined anatomical ROIs according to the Harvard-Oxford (H-O) probabilistic atlas (see Methods in the main text) which included two spatially extensive ROIs (OFC and Frontal Pole). Given our a priori interest in reward and risk representations in prefrontal cortex, the large size of these ROIs, and work documenting functional heterogeneity of these regions, particularly with respect to reward signals [1, 2], we sought to further investigate which sub-region(s) of each of these two large ROIs drove the originally reported decoding effects respective to that ROI. For example, as we were originally able to decode Outcome, E.Risk, RiPE, and RePE in OFC, we probed here which sub-regions of OFC were driving the decoding of those variables. Following our original approach to defining ROIs, to remain free of bias in our sub-regional ROI definition we relied on an independently derived atlas produced by a connectivity-based parcellation [3] of the OFC (Figure S8A) and Frontal Pole (Figure S8B). Within each ROI, we report the number of ROI contacts that spatially comprise each sub-division as determined by the Neubert atlas (see Supplementary Table 2).

We took two analytic approaches to probe the sub-regional contributions to our reported decoding effects in OFC and Frontal Pole. First, we conducted a 'model lesion' analysis in which we dropped the features of the decoding model corresponding to the contacts within a given sub-region (e.g. Area 13 from the Neubert atlas) and measured the change in decoding accuracy compared to a 'full' decoding model containing all contacts in the original large ROI (e.g. OFC from the H-O atlas). The rationale was that the extent to which that sub-region contributed to our originally reported decoding effects would be reflected by the drop in model performance between the nested and full model. In contrast, sub-regions that were not relevant for the decoding of a particular computational variable in that large ROI originally would exhibit relatively less change in model performance upon their exclusion. Our second approach, independent from the first and from the original decoding analysis using the larger H-O ROI, was to re-run a decoding analysis using only the contacts from each sub-region, asking which sub-region(s) of a larger ROI could decode each computational variable, and when in the epoch they did so.

In OFC we were able to decode the reward variables Outcome and RePE in sub-regions 47o (C_{AUC} = 0.386, C_{thresh} = 0.224, p = 0.009, $AUC_{0.263}$ = 0.557, time [0.257-0.311 sec]) and Area 11 (C_{AUC} = 1.189, C_{thresh} = 0.336, p < 0.001, $AUC_{0.319}$ = 0.648, time [0.287-0.335 sec]), respectively, two sub-regions of lateral OFC (Figure S8A). In contrast, activity in central Area 13 could significantly decode both risk-related variables: E.Risk (C_{AUC} = 0.432, C_{thresh} = 0.250, p = 0.013, $AUC_{0.307}$ = 0.563, time [0.281-0.327 sec]), and RiPE (C_{AUC} = 0.598, C_{thresh} = 0.162, p = 0.002, $AUC_{0.222}$ = 0.556, time = [0.196-0.283 sec]; Figure S9A). Notably, we found that the temporal window within which we were able to decode each computational variable from each corresponding OFC sub-region aligned with the temporal windows originally reported using the larger OFC ROI, which in conjunction with convergent results from our 'model lesion' approach (Figure S9B), substantiates the interpretation that these respective sub-regions supported our originally reported OFC decoding effects.

Consistent with the implication of 47o within our original OFC ROI in outcome decoding reported above, we found that more anterior aspects of 47o within our original Frontal Pole ROI was also able to decode outcome (C_{AUC} = 0.330, C_{thresh} = 0.248, p = 0.027, $AUC_{0.273}$ = 0.573) within a similar period [0.250-0.285 sec]. Further, outcome decoding within Frontal Pole was also driven by sub-region 9m, in which outcome could be decoded in early (C_{AUC} = 0.491, C_{thresh} = 0.398, p = 0.032, $AUC_{0.281}$ = 0.579, time [0.248-0.299]) and late (C_{AUC} = 0.499, C_{thresh} = 0.398, p = 0.032, $AUC_{0.409}$ = 0.578, time [0.397-0.457]) temporal clusters. Our Frontal Pole model lesion analysis conditioned on the timing of our original results did not show specificity to 47o or 9m (Figure S9D); instead, multiple sub-regions contributed to our original effect despite most not individually able to decode outcome. Indeed, this is corroborated by the spatial extent of relevant contacts for outcome decoding across our original Frontal Pole ROI (Figure S9E), and together supports the idea that outcome is held in a multivariate, distributed representation across Frontal Pole.

ROI	Sub-region	# Subs	# Contacts
OFC	Area 11	6	11
	Area 13	9	37
	Area 25	3	3
	14m	1	1
	47m	1	1
	47o	9	19
Frontal Pole	Area 11	4	5
	Area 13	3	4
	FPm	2	3
	FPl	8	53
	8m	3	12
	9m	5	9
	11m	5	11
	47m	8	36
	47o	6	13
	Undefined	5	12

Supplementary Table 2: Sub-regional division of OFC and Frontal Pole. # Subs denotes the sample size of subjects going into each sub-region. # Contacts is the number of recording sites per sub-region. Certain sub-region labels are delineated within both OFC and Frontal Pole ROIs (e.g. Area 11). In this case contacts are labeled hierarchically (H-O ROI / Neubert sub-ROI; e.g. OFC / Area 11 versus Frontal Pole / Area 11). Importantly, each contact is only included in one unique sub-division. In other words, there are no overlapping contacts across decoding models. The "undefined" label refers to contacts originally within the Frontal Pole ROI (H-O atlas) but are not within any sub-ROI label of the Neubert atlas; they were not included in analyses.

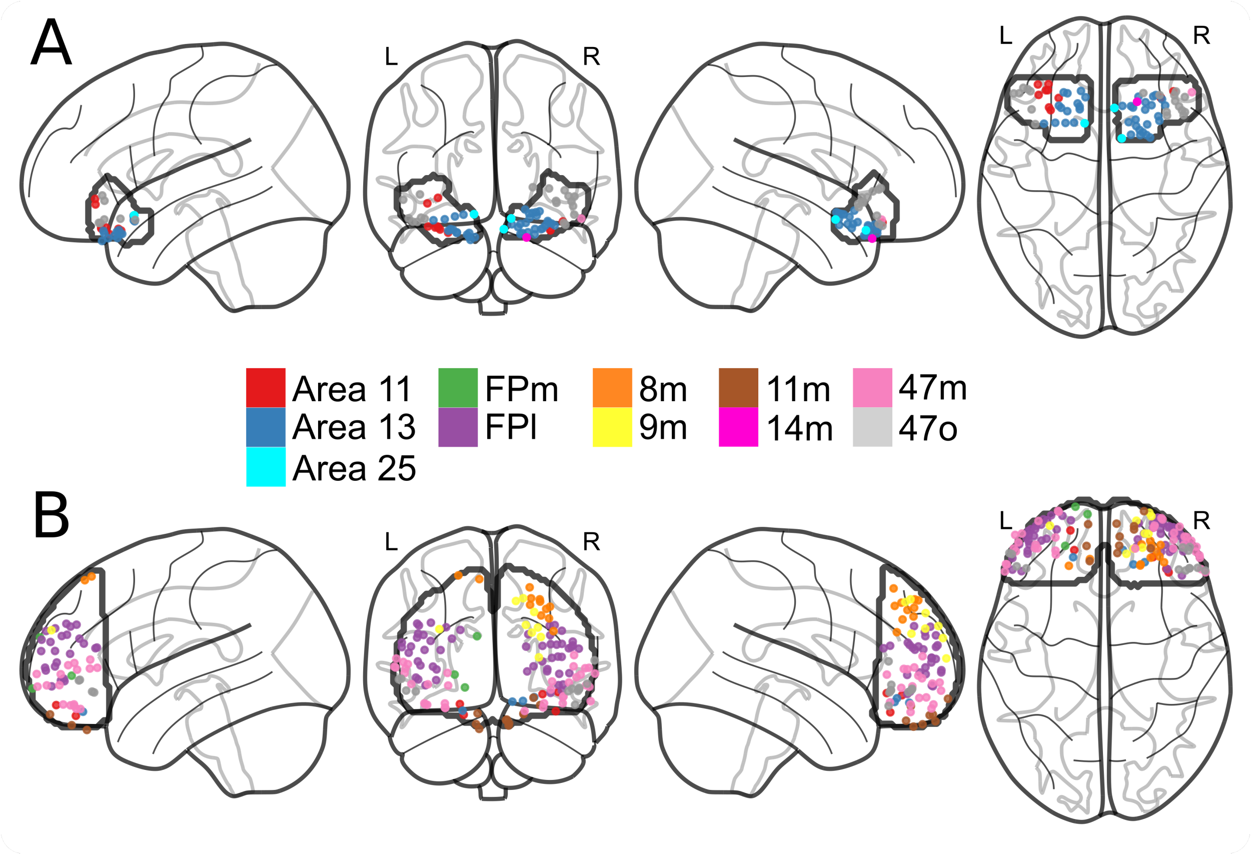

Supplementary Figure 8: Contacts locations in sub-regions of (A) OFC, and (B) Frontal Pole, distinguished by labels from the Neubert atlas [3]. Different colours identify distinct subregions, and contours show boundaries of the H-O atlas ROI. Copyright (c) 2007 - 2023 The nilearn developers. All rights reserved. https://github.com/nilearn/nilearn. Source data are provided as a Source Data file.

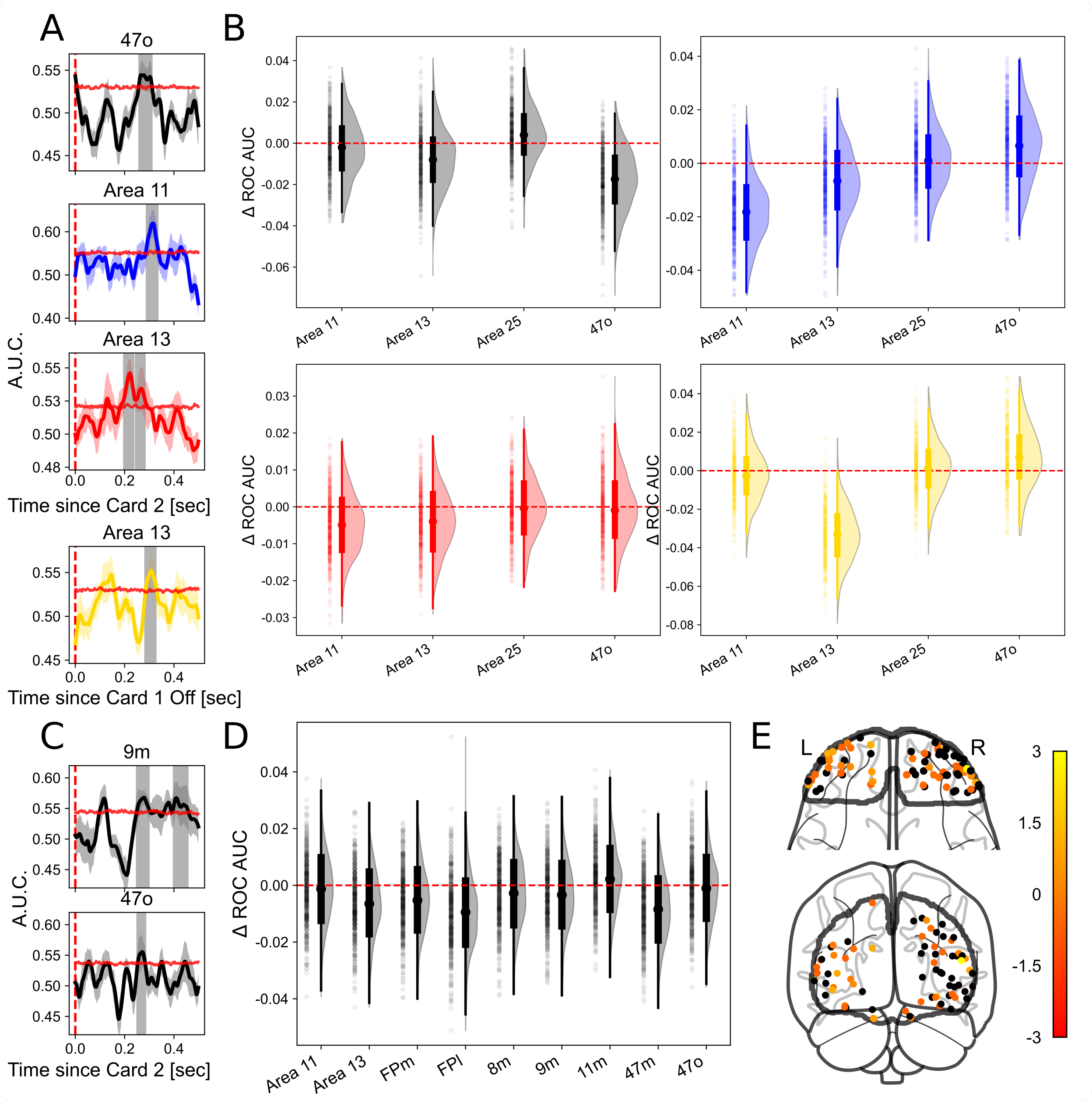

Supplementary Figure 9: Sub-regional contributions to originally reported decoding results in OFC and Frontal Pole. (A) Specific sub-regions of OFC contribute to the decoding of distinct computational variables (black: Outcome; blue: RePE; yellow; E.Risk, red: RiPE). (B) Δ ROC AUC represents the difference between the decoding accuracy (ROC AUC) of each OFC sub-region and the larger OFC ROI, averaged around the significant period respective to each variable reported in the main findings. 95 CI of the bootstrapped test accuracy (shown in the violin and scatter distributions) are depicted with vertical lines, boxes depict the interquartile range, and points represent the median. (C-D) Same as A and B, for Frontal Pole sub-regions. (E) Feature importance map of Frontal Pole shows a spatially distributed outcome representation across sub-regions. Each coloured dot represents a bipolar source which contributed to outcome decoding in Frontal Pole, with colour depicting normalized feature weight. Black dots depict contacts with negative feature weights prior to normalization. Copyright (c) 2007 - 2023 The nilearn developers. All rights reserved. https://github.com/nilearn/nilearn. Source data are provided as a Source Data file.

Supplementary Note 2: Lateralization analysis

For our primary decoding analysis we pooled across left and right hemisphere analogs of a given ROI (e.g. L and R amygdala) to increase the statistical power of our analyses, particularly in ROIs for which we for which we had relatively fewer numbers of contacts (see Supplementary Table 1). Here we interrogate further the relative contribution of left versus right hemisphere contracts for a given ROI in our reported decoding results. Given the limited number of contacts that remain in a ROI when considering only one hemisphere, we provide a descriptive rather than inferential analysis of whether our reported effects were driven primarily by one hemisphere or relied on information provided bilaterally. The logic of this follow-up analysis was that if a reported effect is driven by primarily one hemisphere, then decoding a particular variable from only contacts in that hemisphere would be expected tot yield similar effects. If on the other hand a reported effect relied on bilateral information, decoding that variable only from one hemisphere would result in a relatively lower accuracy score.

For this follow up analysis, we excluded the vmPFC and cingulate gyrus ROIs because they were situated at the midline, and the putamen and supramarginal gyrus because contacts were already lateralized to the right hemisphere (see Supplementary Table 1). While there were 2/30 contacts in the left supramarginal gyrus, because of our bipolar referencing scheme this meant we would only have one neural feature for decoding from left supramarginal gyrus preventing a comparable multivariate analysis. The remaining ROIs were separated into left and right sub-ROIs. Because the purpose of this analysis was to dissect the contribution of sub-regions to our reported findings, we examined the decoding accuracy of the (lateralized) sub-sample of contacts in a given ROI for relevant variables during the period previously reported to be significant. To the extent that one hemisphere is primarily driving the reported effect, we expected to see differential decoding accuracy between left and right ROIs (compared to the bilateral effect reported in the main text) during this period. While we did not conduct statistical tests (e.g. t-tests or non-parametric equivalents such) given the aforementioned limitation of low sample sizes upon dividing bilateral ROIs into left and right regions, we conducted a bootstrap analysis to provide 95% confidence interval estimates of uncertainty using the approach described in the Methods section of the main text.

Using this descriptive approach, we found that the majority of the effects we previously reported were driven bilaterally for each corresponding ROI (Figure S10), with a few potential exceptions. Amygdala contribution to outcome decoding was predominantly driven by the L amygdala (95 CI [-0.127 -0.005], Δ ROC AUC relative to bilateral amygdala), and RiPE decoding in anterior insula showed sensitivity, albeit weaker, to L anterior insula (95 CI [-0.095 0.005], Δ ROC AUC relative to bilateral anterior insula).

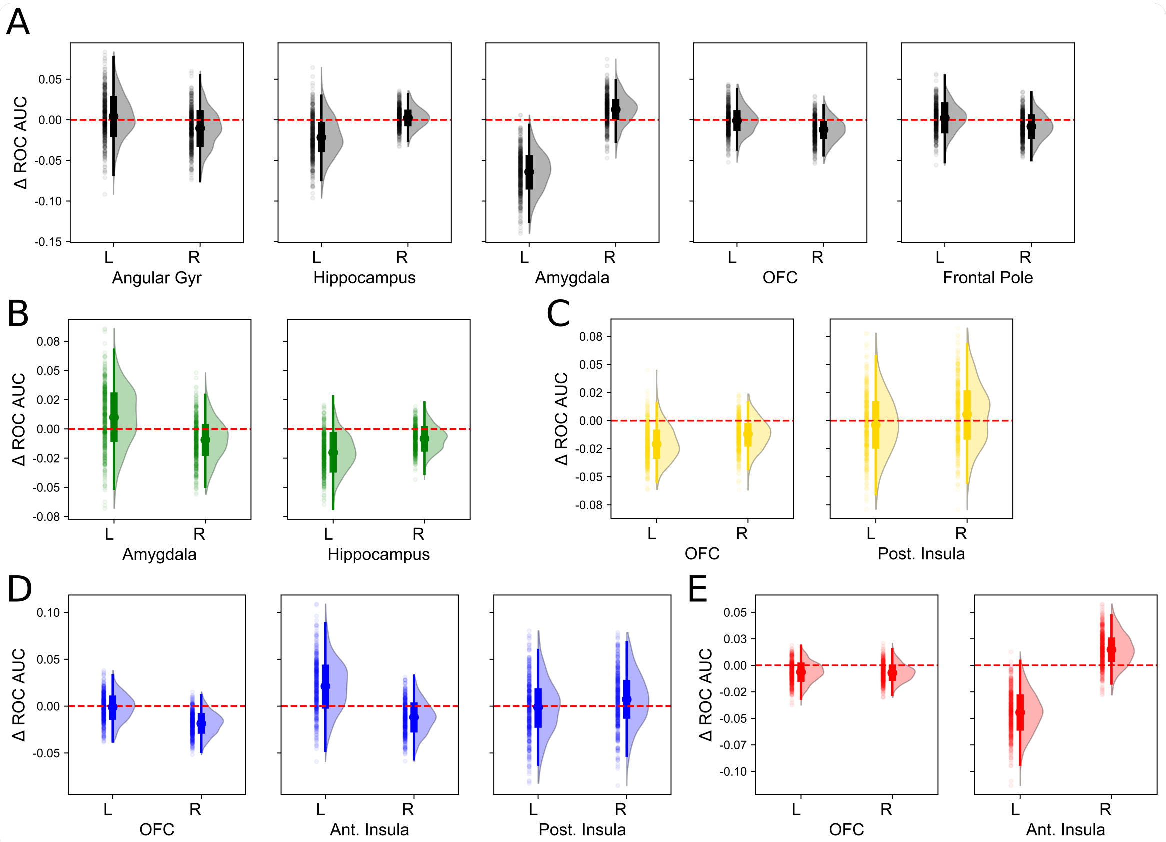

Supplementary Figure 10: Differential contributions of left and right hemisphere ROIs to decoding: A) outcome, B) expected value, C) expected risk, D) reward prediction error, E) risk prediction error. Δ ROC AUC represents the difference between the decoding accuracy (ROC AUC) of left and right subsets of each ROI and the originally reported bilateral ROI (centered at 0; dashed red line), averaged around the significant period respective to each variable and ROI reported in the main findings. 95 CI of the bootstrapped test accuracy (shown in the violin and scatter distributions) are depicted with vertical lines, boxes depict the interquartile range, and points represent the median. Source data are provided as a Source Data file.

Supplementary Note 3: Cross-correlation analysis

We investigated whether neural activity was correlated across regions of interest by running a shuffle-corrected cross-correlation analysis [4] on the neural activity between two regions, following the procedure described in [5] but as applied to iEEG data. We specifically examined whether the average cross-correlation between ROIs was moderated by our computational variables of interest. We followed a feature preprocessing procedure (described in the Methods section of the main text) to regress out of the neural data variables other than the current moderating variable of interest, and normalized the neural data for each contact. We then computed the cross-correlation between the activity in pairs of contacts between regions A and B recorded from the same participant according to:

$$C^r\tau = \sum_{-\infty}^{\infty} S_A^r(t) \cdot S_B^r(t+\tau) \equiv S_A^r \otimes S_B^r \tag{1}$$

where S_A^r, S_B^r denotes the activity from contacts in ROIs A and B, respectively, across the relevant epoch on trial r. We defined the shuffle-corrected cross-correlogram as:

$$V = < S_A^r \otimes S_B^r > - < S_A^r > \otimes < S_B^r > \tag{2}$$

where the $< >$ operator denotes averaging over trials, separately for across-trials splits according to low and high values of the computational variable of interest (following the same approach for binarization described in the main text). This procedure of regularizing the cross-correlogram with the shuffle-correcting term is equivalent to permutation-based approaches (e.g. shift predictor; see [4, 6]) in correcting for time-locked covariation (e.g. caused by stimulus presentation) and reflected in each contact separately. In other words, the shuffle-corrector ensures that if S_A and S_B are independent, the expected value of V is zero. We transformed the resulting cross-correlations using Fisher's r-to-z transformation prior to averaging across pairs [7]. We summed the average z-values separately for high and low trials of the variable, and for negative and positive lag regions of the resulting cross-correlogram. For statistical analyses, we used a two-way ANOVA to test for the interaction between levels of the computational variable of interest (high / low) and lag regions in the space of the cross-correlogram (positive / negative). We also conducted t-tests at each lag region to examine differences in the average cross-correlation between high and low levels of the computational variable of interest.

For each computational variable of interest, we defined pairs of ROIs to submit to the cross-correlation analysis based on regions in which we could decode each respective variable in our main results. In other words, we directed and constrained our cross-correlation analysis as a follow-up to our decoding findings, with the hypothesis that the temporal profile of decoding across regions, for a given variable, would be related to the cross-correlation of signals across those regions. Specifically, we hypothesized that areas earlier in the temporal profile of a variable (as informed by the results of our decoding analyses) would predict delayed signals in areas later in the timing profile of that variable. For EV, Outcome, and E.Risk, we aligned the ROIs based on their peak decoding accuracy and tested for differences in the cross-correlation between adjacent regions (e.g. for outcome representations: Angular Gyrus and Hippocampus, Hippocampus and Amygdala, etc) as a function of trials sorted along high or low levels of the variable. For RePE and RiPE, we defined pairs of ROIs for cross-correlation analysis based on the temporal profile of decoding common to these two variables, in which RePE and RiPE are first decoded in one ROI, followed by parallel decoding across a set of regions. For these latter two variables we thus computed cross-correlations between a seed ROI in which we found early decoding (RePE: Cingulate gyrus; RiPE: OFC), and each target ROI in which we could decode the variable relatively later.

Across all computational variables and their respective pairs of ROIs, we did not find significant interactions between lag and variable level in the cross-correlation measure (see Supplementary Tables 3-7). When we examined differences in cross-correlation between high and low levels of the computational variable separately at positive and negative lags, we found that there were different correlations between early signal in the frontal pole and later signal in OFC across observed outcomes ($t(334) = -2.226$, $p = 0.027$). Similarly, early activity in OFC contacts predicted later vmPFC activity in an outcome-dependent manner ($t(24) = 2.165$, $p = 0.041$; Figure S11C). However as the interaction tests respective to each of these simple effects were not significant, we refrain from making more interpretations about directed connectivity or causal relationships between regions.

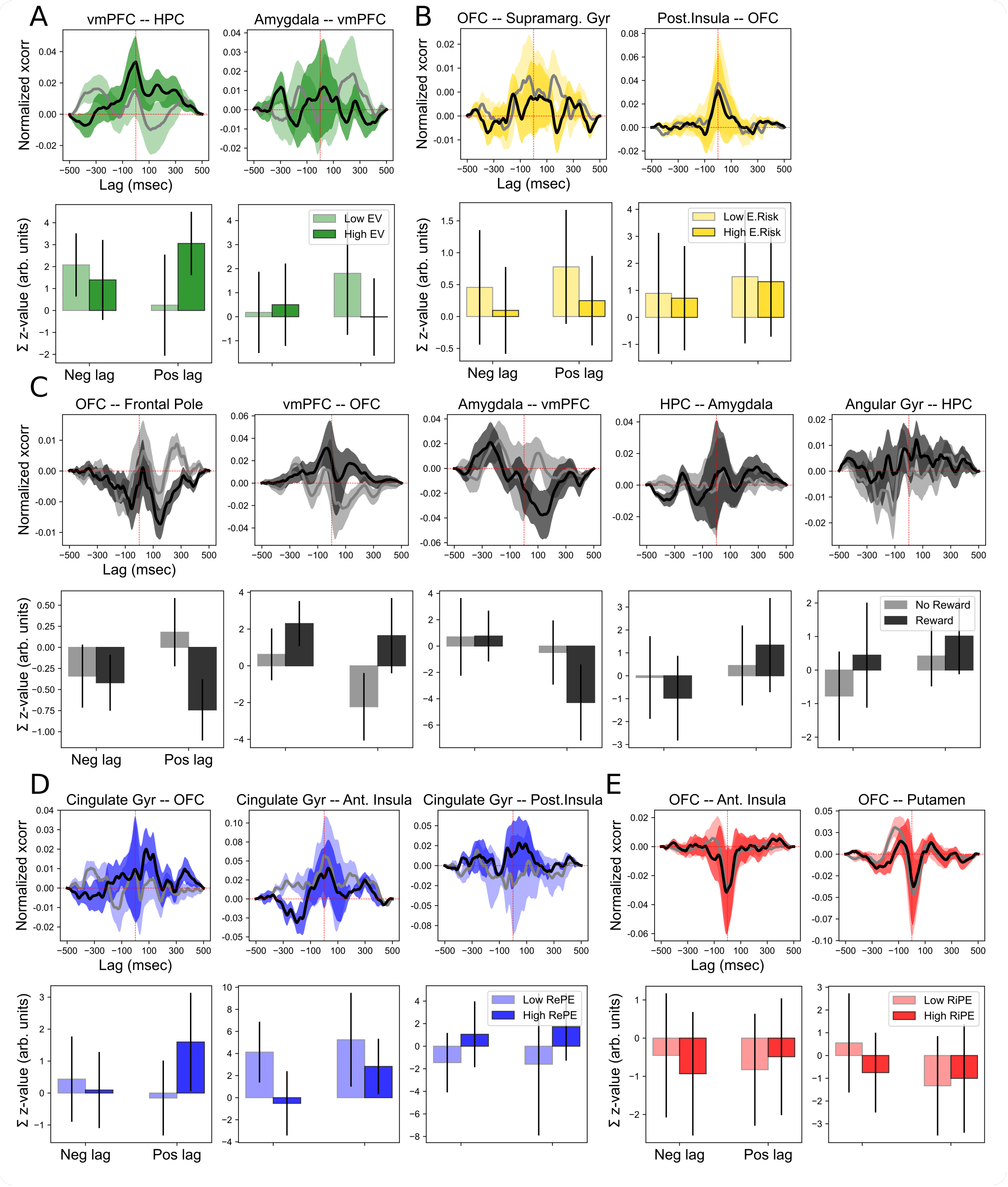

Supplementary Figure 11: Cross-correlation analysis between pairs of ROIs respective to the decoding results for each variable: A) EV, B) E.Risk, C) Outcome, D) RePE, E) RiPE. Top rows in each panel depict the shuffle-corrected cross-correlogram with traces and shaded areas depicting the mean and standard error normalized cross-correlation over across-ROI contact pairs. The bottom rows depict a summary of the cross-correlogram calculated by integrating over the area under each mean trace, separately for low and high trials of the respective variable and for negative and positive lags. Source data are provided as a Source Data file.

Seed ROI	Target ROI	Interaction	Neg. Lag	Pos. Lag
vmPFC	HPC	F(1,4) = 2.850, p = 0.167	t(19) = -0.381, p = 0.707	t(19) = 0.965, p = 0.347
Amygdala	vmPFC	F(1,2) = 0.251, p = 0.666	t(11) = 0.160, p = 0.876	t(11) = -0.824, p = 0.427

Supplementary Table 3: Cross-correlation between temporally adjacent ROIs for expected value. Omnibus statistics are calculated from a repeated-measures analysis of variance (ANOVA) test, and simple effects are calculated from a two-sided paired t-test.

Seed ROI	Target ROI	Interaction	Neg. Lag	Pos. Lag
OFC	Frontal Pole	F(1,7) = 0.688, p = 0.434	t(334) = -0.190, p = 0.849	t(334) = -2.226, **p = 0.027**
vmPFC	OFC	F(1,4) = 4.493, p = 0.101	t(24) = 1.306, p = 0.204	t(24) = 2.165, **p = 0.041**
Amygdala	vmPFC	F(1,2) = 2.407, p = 0.261	t(11) = 0.019, p = 0.985	t(11) = -1.231, p = 0.244
HPC	Amygdala	F(1,7) = 2.686, p = 0.145	t(35) = -0.757, p = 0.454	t(35) = 0.483, p = 0.632
Angular Gyr	HPC	F(1,4) = 0.389, p = 0.567	t(24) = 0.582, p = 0.566	t(24) = 0.470, p = 0.642

Supplementary Table 4: Cross-correlation between temporally adjacent ROIs for outcome. Simple effects in which $p < 0.05$ are bolded, but their respective interaction terms are not statistically significant. Omnibus statistics are calculated from a repeated-measures analysis of variance (ANOVA) test, and simple effects are calculated from a two-sided paired t-test.

Seed ROI	Target ROI	Interaction	Neg. Lag	Pos. Lag
OFC	Supramarg. Gyr	F(1,5) = 0.401, p = 0.554	t(60) = -0.391, p = 0.697	t(60) = -0.562, p = 0.576
Post.Insula	OFC	F(1,4) = 0.514, p = 0.513	t(17) = -0.119, p = 0.906	t(17) = -0.114, p = 0.911

Supplementary Table 5: Cross-correlation between temporally adjacent ROIs for expected risk. Omnibus statistics are calculated from a repeated-measures analysis of variance (ANOVA) test, and simple effects are calculated from a two-sided paired t-test.

Seed ROI	Target ROI	Interaction	Neg. Lag	Pos. Lag
Cingulate Gyr	OFC	F(1,5) = 0.106, p = 0.758	t(19) = -0.198, p = 0.845	t(19) = 1.408, p = 0.175
Cingulate Gyr	Ant. Insula	F(1,2) = 0.737, p = 0.481	t(2) = -3.339, p = 0.079	t(2) = -0.613, p = 0.602
Cingulate Gyr	Post.Insula	F(1,2) = 0.015, p = 0.914	t(2) = 0.534, p = 0.647	t(2) = 0.930, p = 0.450

Supplementary Table 6: Cross-correlation between temporally adjacent ROIs for reward prediction error. Omnibus statistics are calculated from a repeated-measures analysis of variance (ANOVA) test, and simple effects are calculated from a two-sided paired t-test.

Seed ROI	Target ROI	Interaction	Neg. Lag	Pos. Lag
OFC	Ant. Insula	F(1,5) = 2.550, p = 0.171	t(25) = -0.445, p = 0.660	t(25) = 0.263, p = 0.795
OFC	Putamen	F(1,3) = 1.634, p = 0.291	t(13) = -0.841, p = 0.415	t(13) = 0.193, p = 0.85

Supplementary Table 7: Cross-correlation between temporally adjacent ROIs for risk prediction error. Omnibus statistics are calculated from a repeated-measures analysis of variance (ANOVA) test, and simple effects are calculated from a two-sided paired t-test.

References

1. Wallis, J. D. & Kennerley, S. W. Heterogeneous reward signals in prefrontal cortex. *Current opinion in neurobiology* **20,** 191–198 (2010).
2. Kringelbach, M. L. & Rolls, E. T. The functional neuroanatomy of the human orbitofrontal cortex: evidence from neuroimaging and neuropsychology. *Progress in neurobiology* **72,** 341–372 (2004).
3. Neubert, F.-X., Mars, R. B., Sallet, J. & Rushworth, M. F. Connectivity reveals relationship of brain areas for reward-guided learning and decision making in human and monkey frontal cortex. *Proceedings of the national academy of sciences* **112,** E2695–E2704 (2015).
4. Brody, C. D. Correlations without synchrony. *Neural computation* **11,** 1537–1551 (1999).
5. Aquino, T. G., Courellis, H. S., Mamelak, A., Rutishauser, U. & O'Doherty, J. Encoding of predictive associations in human prefrontal and medial temporal neurons during Pavlovian conditioning. *bioRxiv,* 2023–02 (2023).
6. Perkel, D. H., Gerstein, G. L. & Moore, G. P. Neuronal spike trains and stochastic point processes: II. Simultaneous spike trains. *Biophysical journal* **7,** 419–440 (1967).
7. Silver, N. C. & Dunlap, W. P. Averaging correlation coefficients: should Fisher's z transformation be used? *Journal of applied psychology* **72,** 146 (1987).
