# Supplementary material for: Temporally organized representations of reward and risk in the human brain
Source: Nat Commun. 2024 Mar 9;15:2162. doi: 10.1038/s41467-024-46094-1 (PMC10924934; doi:10.1038/s41467-024-46094-1)
Supplement: Supplementary file 3 — Reporting Summary [file 41467_2024_46094_MOESM3_ESM.pdf]

nature portfolio

Corresponding author(s): Vincent Man
Last updated by author(s): Jan 17, 2024

Reporting Summary

Nature Portfolio wishes to improve the reproducibility of the work that we publish. This form provides structure for consistency and transparency in reporting. For further information on Nature Portfolio policies, see our Editorial Policies and the Editorial Policy Checklist.

Statistics

For all statistical analyses, confirm that the following items are present in the figure legend, table legend, main text, or Methods section.

n/a	Confirmed	
☐	☒	The exact sample size (*n*) for each experimental group/condition, given as a discrete number and unit of measurement
☐	☒	A statement on whether measurements were taken from distinct samples or whether the same sample was measured repeatedly
☐	☒	The statistical test(s) used AND whether they are one- or two-sided<br>*Only common tests should be described solely by name; describe more complex techniques in the Methods section.*
☐	☒	A description of all covariates tested
☐	☒	A description of any assumptions or corrections, such as tests of normality and adjustment for multiple comparisons
☐	☒	A full description of the statistical parameters including central tendency (e.g. means) or other basic estimates (e.g. regression coefficient) AND variation (e.g. standard deviation) or associated estimates of uncertainty (e.g. confidence intervals)
☐	☒	For null hypothesis testing, the test statistic (e.g. *F*, *t*, *r*) with confidence intervals, effect sizes, degrees of freedom and *P* value noted<br>*Give P values as exact values whenever suitable.*
☒	☐	For Bayesian analysis, information on the choice of priors and Markov chain Monte Carlo settings
☐	☒	For hierarchical and complex designs, identification of the appropriate level for tests and full reporting of outcomes
☐	☒	Estimates of effect sizes (e.g. Cohen's *d*, Pearson's *r*), indicating how they were calculated

Our web collection on statistics for biologists contains articles on many of the points above.

Software and code

Policy information about availability of computer code

Data collection	Data were collected using the Psychtoolbox-3 package (v3.0.14, http://psychtoolbox.org) in the MATLAB (R2017a, https://www.mathworks.com/products/matlab.html) environment. Custom code used for data collection is uploaded to a public Github repository: https://github.com/manvincent/temp_org_iEEG
Data analysis	Imaging data were analyzing using FSL (v6.0, https://fsl.fmrib.ox.ac.uk/fsl/fslwiki) and ANTS (v2.3.1, http://stnava.github.io/ANTs) packages. Behavioural and electrophysiological data were analyzed using custom software relying on the MNE package (v1.2.3, https://mne.tools/stable/ index.html) the scikit-learn package (v.1.30, https://scikit-learn.org/stable/) and the Nilearn package (v0.10.0; https://nilearn.github.io/stable/index.html) in the Python (v3.0, https://www.python.org/) environment. Custom code used for analysis is uploaded to a public Github repository: https://github.com/manvincent/temp_org_iEEG

For manuscripts utilizing custom algorithms or software that are central to the research but not yet described in published literature, software must be made available to editors and reviewers. We strongly encourage code deposition in a community repository (e.g. GitHub). See the Nature Portfolio guidelines for submitting code & software for further information.

Data

Policy information about availability of data

All manuscripts must include a data availability statement. This statement should provide the following information, where applicable:

- Accession codes, unique identifiers, or web links for publicly available datasets
- A description of any restrictions on data availability
- For clinical datasets or third party data, please ensure that the statement adheres to our policy

Data and materials from this study are available in the Open Science Framework (OSF) database. Identifier: DOI 10.17605/OSF.IO/RKG4Q. The following databases were used in the study: Harvard-Oxford probabilistic atlas and MNI152 standard space template from the FSL toolbox (v6.0; https://fsl.fmrib.ox.ac.uk/fsl/fslwiki/Atlases), and the Neubert cingulate and orbitofrontal cortex atlas (http://www.rbmars.dds.nl/CBPatlases.htm). Source data are provided with this paper.

Research involving human participants, their data, or biological material

Policy information about studies with human participants or human data. See also policy information about sex, gender (identity/presentation), and sexual orientation and race, ethnicity and racism.

Reporting on sex and gender	Patient sex is reported in the methods section. Sex and gender were not considered in the design of the experiment and sex- and gender-based analyses were not performed due to limited patient availability and sample size. Findings do not speak to sex- or gender-based differences.
Reporting on race, ethnicity, or other socially relevant groupings	Race, ethnicity, or other socially relevant groupings were not considered in the design of the experiment or analyses due to limited patient availability and sample size. Findings do not speak to differences between race, ethnicity, or other socially relevant groupings.
Population characteristics	See below.
Recruitment	Participants were recruited from epilepsy patients ongoing monitoring prior to surgical treatment of refractory epilepsy. Participants needed to meet the inclusion criteria of being over 18 years old, and 3/10 participants were male. Given the sampling biases introduced by the constraints of recruiting from a patient population for intra-cranial recording, these results may have limited generalization beyond the current population. Future studies should examine potential sex and gender differences as well as generalization beyond the age cohort of the present study.
Ethics oversight	University of Iowa Institutional Review Board.

Note that full information on the approval of the study protocol must also be provided in the manuscript.

Field-specific reporting

Please select the one below that is the best fit for your research. If you are not sure, read the appropriate sections before making your selection.

☐ Life sciences ☒ Behavioural & social sciences ☐ Ecological, evolutionary & environmental sciences

For a reference copy of the document with all sections, see nature.com/documents/nr-reporting-summary-flat.pdf

Behavioural & social sciences study design

All studies must disclose on these points even when the disclosure is negative.

Study description	Quantitative experimental data. Behavioural responses to a psychological task paradigm and intra-cranial recordings from human participants.
Research sample	Male and female refractory epilepsy patients from 22-56 years old. This sample was chosen since these patients were already going through seizure monitoring prior to surgical intervention according to clinical criteria. This sample is representative of a population of epileptic patients in the Iowa and Midwestern United States area
Sampling strategy	No explicit sampling strategy was employed. The dataset is consistent with typical data collected for intra-cranial research by our group and others. This sample and dataset size is consistent with similar previously published work (e.g. Domenech et al., 2020, Science; Saez et al., 2018, Curr Bio; Aquino et al., 2020, J Neurosci; Gander et al., 2019, NeuroImage), and allows for enough recording sessions and channels to allow for the decoding analyses reported in the study.
Data collection	Data were collected using a combination of depth macroelectrodes and sub-dural grid electrodes with a Neuralynx ATLAS system. Electrode and contact locations were positioned exclusively according to clinical criteria. The task was performed on a laptop positioned on a table by the patient's bed. The researcher was not blind to the experimental hypothesis when performing the task. Other than the researcher and participant, only the attending surgeon and nurses were present during the study for clinical purposes.

Timing	Data were collected from April 2017 to May 2021.
Data exclusions	All sessions completed by the patients were included. No trial data were excluded; covariates were included to model trials in which participants did not respond correctly for the statistical analyses.
Non-participation	No patients declined participation.
Randomization	Participants were not allocated into experimental groups, and thus no randomization into patient groups. This was not relevant to our study since we were not interested in any hypotheses involving subgroups of patients.

Reporting for specific materials, systems and methods

We require information from authors about some types of materials, experimental systems and methods used in many studies. Here, indicate whether each material, system or method listed is relevant to your study. If you are not sure if a list item applies to your research, read the appropriate section before selecting a response.

Materials & experimental systems

n/a	Involved in the study
☒	☐ Antibodies
☒	☐ Eukaryotic cell lines
☒	☐ Palaeontology and archaeology
☒	☐ Animals and other organisms
☒	☐ Clinical data
☒	☐ Dual use research of concern
☒	☐ Plants

Methods

n/a	Involved in the study
☒	☐ ChIP-seq
☒	☐ Flow cytometry
☒	☐ MRI-based neuroimaging

Plants

Seed stocks	*Report on the source of all seed stocks or other plant material used. If applicable, state the seed stock centre and catalogue number. If plant specimens were collected from the field, describe the collection location, date and sampling procedures.*
Novel plant genotypes	*Describe the methods by which all novel plant genotypes were produced. This includes those generated by transgenic approaches, gene editing, chemical/radiation-based mutagenesis and hybridization. For transgenic lines, describe the transformation method, the number of independent lines analyzed and the generation upon which experiments were performed. For gene-edited lines, describe the editor used, the endogenous sequence targeted for editing, the targeting guide RNA sequence (if applicable) and how the editor was applied.*
Authentication	*Describe any authentication procedures for each seed stock used or novel genotype generated. Describe any experiments used to assess the effect of a mutation and, where applicable, how potential secondary effects (e.g. second site T-DNA insertions, mosiacism, off-target gene editing) were examined.*
